# Supplementary material for: Content-rich biological network constructed by mining PubMed abstracts
Source: BMC Bioinformatics. 2004 Oct 8;5:147. doi: 10.1186/1471-2105-5-147 (PMC528731; doi:10.1186/1471-2105-5-147)
Supplement: Additional File 2 — The original results of the above study (non-essential files are deleted to keep the file size under the limit set by BMC bioinformatics). [file 1471-2105-5-147-S2.bz2 › chilibotAdditionalFile2/dip10/223ID7831290E739/html/SYK_LYN.html]

 


 **SYK** and **LYN** 
  
Found 264 abstracts in PubMed, retrieved 10.  
 

 What does Google say? 
 PDF only 
| .edu only 

---

**Interactive relationship** (e.g. stimulation, inhibition, etc)

**Stimulatory relationship**- Methyl dihydroxycinnamate DHC, a PTK inhibitor, was more selective for  **Syk**  than  **Lyn** , while staurosporine ST, a broad specificity protein kinase inhibitor, inhibited  **Lyn**  more than  **Syk** .  Ref: 12739161 Pflugers Arch, 2003
**Neutral relationship**

**Non-interactive relationship** (e.g. studied together, co-existance, homology, etc.)

- The activity of the kinases that phosphorylate the Fc receptor gamma chain, Fyn and  **Lyn** , as well as the tyrosine kinase  **Syk**  and phosphoinositide 3 kinase was also inhibited by quercetin in a concentration dependent manner, both in whole cells and in isolation.  Ref: 12871380 J Thromb Haemost, 2003
- Under our conditions, hydrogen peroxide induced PI3K and Akt activation was independent of  **Lyn** ,  **Syk** , Cbl, BCAP, or Ras when each was eliminated individually either by mutation or by a specific inhibitor.  Ref: 12627965 Biochemistry, 2003
- In contrast, the tumor cells in all cases of lymphocyte predominance Hodgkin rad s disease were positive for Fyn,  **Syk** , BLNK and PLC gamma2, and  **Lyn**  immunostaining was seen in a minority of biopsies.  Ref: 12881301 BloodBlood, 2003
- This phosphorylation was intact in Btk and Cbl deficient DT40 cells, but was drastically suppressed in  **Lyn** ,  **Syk** , or BCAP deficient DT40 cells.  Ref: 12627965 Biochemistry, 2003
- We have reported previously that deoxygenation of sickle cells stimulates KCC and activates  **Syk**  a  **Syk**  family PTK, but not  **Lyn**  an Src family PTK.  Ref: 12739161 Pflugers Arch, 2003
- Our approach was to build a detailed mathematical model of reactions involving Fc epsilon RI,  **Lyn** ,  **Syk** , and a bivalent ligand that aggregates Fc epsilon RI.  Ref: 12646643 J Immunol, 2003
- It is concluded that EGCG inhibits platelet activation, by hindering the thrombin proteolytic activity, and by reducing the agonist induced Ca 2 c increase through inhibition of  **Syk**  and  **Lyn**  activities.  Ref: 12719785 Thromb Haemost, 2003
- An in vitro binding study revealed that phosphorylation of Tyr 446 by  **Syk**  was likely to create a binding site for the  **Lyn**  SH2 domain in RBL 2H3 cells.  Ref: 12709437 J Biol Chem< MedlineTA>J Biol Chem, 2003
- We investigated the roles of the Src kinase  **Lyn** , the immunoreceptor tyrosine based activation motifs ITAMs on the beta and gamma subunits of Fc epsilon RI, and  **Syk**  itself in the activation of  **Syk** .  Ref: 12646643 J Immunol, 2003
- In addition, interpreted in the context of the model, experimentally observed differences between the beta and gamma chains with respect to levels of phosphorylation and rates of dephosphorylation indicate that most cellular  **Syk** , but only a small fraction of  **Lyn** , is available to interact with receptors.  Ref: 12646643 J Immunol, 2003
- Stable expression of this  **Lyn**  PLCgamma2 was not accompanied by an increase in substrate hydrolysis in resting cells which followed stimulation and specifically required presence and or activation of  **Syk** , Btk, PI 3K but not BLNK, as established using deficient cell lines or specific inhibitors.  Ref: 12780340 Biochem J< MedlineTA>Biochem J, 2003
- Both tyrosine kinases  **Syk**  and  **Lyn** , immuno precipitated from stimulated platelets, are greatly inhibited upon cellular pre incubation with EGCG, which also inhibits the in vitro auto phosphorylation and exogenous activity of these two enzymes purified from rat spleen.  Ref: 12719785 Thromb Haemost, 2003
